# Protein Function Prediction Using Deep Restricted Boltzmann Machines

**DOI:** 10.1155/2017/1729301

**Published:** 2017-06-28

**Authors:** Xianchun Zou, Guijun Wang, Guoxian Yu

**Affiliations:** College of Computer and Information Science, Southwest University, Chongqing, China

## Abstract

Accurately annotating biological functions of proteins is one of the key tasks in the postgenome era. Many machine learning based methods have been applied to predict functional annotations of proteins, but this task is rarely solved by deep learning techniques. Deep learning techniques recently have been successfully applied to a wide range of problems, such as video, images, and nature language processing. Inspired by these successful applications, we investigate deep restricted Boltzmann machines (DRBM), a representative deep learning technique, to predict the missing functional annotations of partially annotated proteins. Experimental results on* Homo sapiens*,* Saccharomyces cerevisiae*,* Mus musculus,* and* Drosophila* show that DRBM achieves better performance than other related methods across different evaluation metrics, and it also runs faster than these comparing methods.

## 1. Introduction

Proteins are the major components of living cells, they are the main material basis that form and maintain life activities. Proteins engage with various biological activities, such as catalysis of biochemical reactions and transport to signal transduction [1, 2]. High-throughput biotechniques produce explosive growth of biological data. Due to experimental techniques and the research bias in biology [3, 4], the gap between newly discovered genome sequences and functional annotations of these sequences is becoming larger and larger. The Human Proteome Project consortium recently claimed that we still have very little information about the cellular functions of approximately two-thirds of human proteins [5]. Wet-lab experiments can precisely verify functions of proteins, but it is time consuming and costly to do so. In practice, wet-lab techniques can only verify a portion of functions of proteins. In addition, it is difficult to efficiently verify functional annotations of massive proteins by wet-lab techniques. Therefore, it is important and necessary to develop computational models to make use of available functional annotations of proteins and a variety of types genomic and proteomic data, to automatically infer protein functions [2, 6].

Various computational methods have been proposed to predict functional annotations of proteins. These methods are often driven by data-intensive computational models. Data may come from amino acids sequences [7], protein-protein interactions [8], pathways [9], and multiple types of biological data fusion [10–12]. Gene Ontology (GO) is a major bioinformatics tool to unify gene products' attributes across all species, it uses GO terms to describe the gene products attributes [13], and these terms are structured in a directed acyclic graph (DAG). Each GO term in the graph can be viewed as a functional label and is associated with a distinct alphanumeric identifier, that is, GO:0008150 (biological process). GO is not static. Researchers and GO consortium contribute to updating GO as the revolved biological knowledge. Currently, most functional annotations of proteins are shallow and far from complete [3–5]. Given the true path rule of GO [13], if a protein is annotated with a GO term, then all the ancestor terms of that term are also annotated to the protein, but it is uncertain whether its descendant terms should be annotated to the protein or not. Therefore, it is more desirable to know the specific annotations of a protein, rather than the general ones, and the corresponding specific terms can provide more biological information than the shallow ones, which are ancestor terms of these specific terms. In this work, we investigate to predict deep (or specific) annotations of a protein based on the available annotations of proteins.

Functional associations between proteins and GO structure have been directly employed to predict protein functions [14–18]. Functional annotations of proteins can be encoded by a protein function association matrix, in which each row corresponds to a protein and each column represents a type of function. King et al. [14] directly used decision tree classifier (or Bayes classifier) on the pattern of annotations to infer additional annotations of proteins. But these two classifiers need sufficient annotations and they get rather poor performance on specific GO terms, which are annotated to fewer than 10 proteins. Khatri et al. [15] used truncated single value decomposition (tSVD) to replenish the missing functions of proteins based on protein function matrix. This approach is able to predict missing annotations in existing annotation databases and improve prediction accuracy. But this method does not take advantage of the hierarchical and flat relationships between GO terms. Previous researches have demonstrated that the ontology hierarchy plays important roles in predicting protein function [2, 16, 18]. Done et al. [16] used a vector space model and a number of weighting schemes, along with latent semantic indexing approach to extract implicit semantic relationships between proteins and those between functions to predict protein functions. This method is called NtN [16]. NtN takes into account GO hierarchical structure and can weigh different GO terms situated at different locations of GO DAG [19]. Tao et al. [17] proposed a method called information theory based semantic similarity (ITSS). ITSS first calculates the semantic similarity between pairwise GO terms in a hierarchy and then sums up these pairwise similarity for pairwise GO terms annotated to two proteins. Next, it uses a *k*NN classifier to predict novel annotations of a protein. Yu et al. [18] proposed downward random walks (dRW) to predict missing (or new) functions of partially annotated proteins. Particularly, dRW applies downward random walks with restart [20] on the GO DAG, started on terms annotated to a protein, to predict additional annotations of the protein.

A protein is often engaged with several biological activities and thus is annotated with several GO terms. Each term can be regarded as a functional label, and protein function prediction can be modeled as a multilabel learning problem [21, 22]. From this viewpoint, protein function prediction using incomplete annotations can be modeled as a multilabel weak learning problem [22]. More recently, Yu et al. [23] proposed a method called PILL to replenish missing functions for partially annotated proteins using incomplete hierarchical labels information. Fu et al. [24] proposed a method called dHG to predict novel functions of proteins using a directed hybrid graph, which is consisted with GO DAG, protein-protein interaction network, and available functional associations between GO terms and proteins. These aforementioned methods (except DRBM) can be regarded as shallow machine learning approaches [25]. They do not capture deep associations between proteins and GO terms.

In this paper, we investigate the recently widely applied technique, deep learning [25], to capture deep associations between proteins and GO terms, and to replenish the missing annotations of incompletely annotated proteins. For this investigation, we apply deep restricted Boltzmann machines (DRBM) to predict functional annotations of proteins. DRBM utilizes the archived annotations of four model species (*Homo sapiens*,* Saccharomyces cerevisiae*,* Mus musculus,* and* Drosophila*) to explore the hidden associations between proteins and GO terms and the structural relationship between GO terms. At the same time, it optimizes the parameters of DRBM. After that, we validate the performance of DRBM by comparing its predictions with recently archived GO annotations of these four species. The empirical and comparative study shows DRBM achieves better results than other related methods. DRBM also runs faster than some of these comparing methods.

The structure of this paper is organized as follows.  Section 2 briefly reviews some related deep learning techniques that are recently applied for protein function prediction.  Section 3 introduces the restricted Boltzmann machine and deep restricted Boltzmann machine for protein function prediction. The experimental datasets, setup, and results are discussed in Section 4. Conclusions are provided in Section 5.

## 2. Related Work

Some pioneers have already applied deep learning for some bioinformatics problems [26], but few works have been reported for protein function prediction. Autoencoder neural networks (AE) can process complex structural data better than shallow machine learning methods [25, 27, 28]. AE has been applied in computer vision [28], speech recognition [25, 27], and protein residue-residue contacts prediction [26]. Chicco et al. [29] recently used deep AE to predict protein functions. Experiments show that deep AE can explore the deep associations between proteins and GO terms and achieve better performance than other shallow machine learning based function prediction methods, including tSVD [29].

Deep AE takes much more time in fine-tuning network; if the network is very deep, it will lead to vanishing gradient problem. In this work, we suggest to use deep restricted Boltzmann machines (DRBM), instead of AE, to predict functional annotations of proteins. DRBM has rapid convergence speed and good stability. DRBM has been used to construct the deep belief networks [30], for speech recognition [31, 32], collaborative filtering [33], computational biology [34], and other fields. Recently, Wang and Zeng [34] proposed to predict drug-target interactions using restricted Boltzmann machines and achieved good prediction performance. More recently, Li et al. [35] used conditional restricted Boltzmann machines to capture high-order label dependence relationships and facilitate multilabel learning with incomplete labels. Experiments have demonstrated the efficacy of restricted Boltzmann machines on addressing multilabel learning with incomplete labels.

To the best of our knowledge, few teams investigate DRBM for large-scale missing functions prediction. For this purpose, we study it for predicting functions of proteins of* Homo sapiens*,* Saccharomyces cerevisiae*,* Mus musculus*, and* Drosophila* and compare it with a number of related methods. The experimental results show that DRBM achieves better results than these comparing methods on various evaluation metrics.

## 3. Methods

In this section, we will describe the deep restricted Boltzmann machines to predict missing GO annotations of proteins.

### 3.1. Restricted Boltzmann Machine

**Figure 1:**
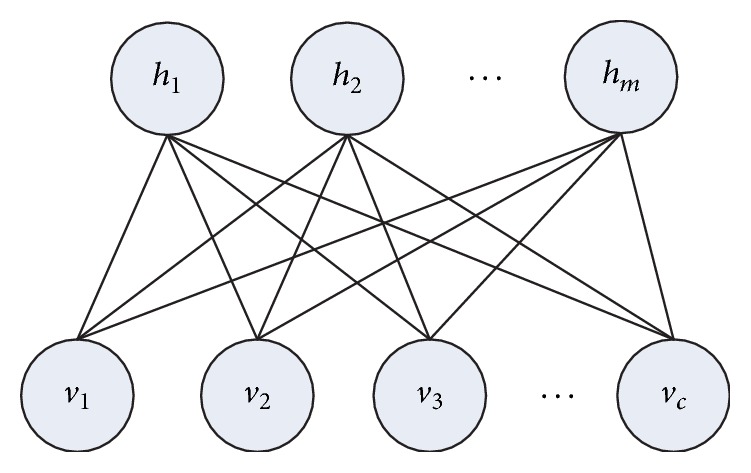
An RBM with binary hidden units (*h*_*j*_) representing latent features and visible units (*v*_*i*_) encoding observed data.

A restricted Boltzmann machine (RBM) is a network of undirected graphical model with stochastic binary units [32]. As shown in Figure 1, an RBM is a two-layer bipartite graph with two types of units, a set of visible units *v* ∈ {0,1}, and a set of hidden units *h* ∈ {0,1}. Input units and hidden units are fully connected; there is no connection between nodes in the same layer. In this paper, the number of visible units is equal to the number of GO terms, and these units take the protein function association matrix as inputs.

RBM is an unsupervised method; it learns one layer of hidden features. When the number of hidden units is smaller than that of visual units, the hidden layer can deal with nonlinear complex dependency and structure of data, capture deep relationship from input data [30], and represent the input data more compactly. Latent feature values are represented by the hidden units and visible units encode available GO annotations of proteins. Suppose there are *c* (the number of GO terms) visible units and *m* hidden units in an RBM. *v*_*i*_  (*i* = 1,…, *c*) indicates the state of the *i*th visible unit, where *v*_*i*_ = 1 means the *i*th term is annotated to the protein and *v*_*i*_ = 0 means the *i*th term is not associated with the protein. Binary variable *h*_*j*_  (*j* = 1,…, *m*) indicates the state of hidden unit, and *h*_*j*_ = 1 denotes the *j*th hidden unit which is active. Let *W*_*ij*_ be the weight associated with the connection between *v*_*i*_ and *h*_*j*_. (*v*, *h*) is a joint configuration of an RBM.

The energy function capturing the interaction patterns between visual layer and hidden layer can be modeled as follows:

(1) $$\begin{array}{c} E\left(\;v,h\;|\;\theta \;\right)=-\overset{C}{\underset{i=1}{\sum }}a_{i}v_{i}-\overset{m}{\underset{j=1}{\sum }}b_{j}h_{j}-\overset{C}{\underset{i=1}{\sum }}\;\overset{m}{\underset{j=1}{\sum }}v_{i}W_{ij}h_{j}, \end{array}$$

where *θ* = {*W*_*ij*_, *a*_*i*_, *b*_*j*_} are parameters of RBM, while *a*_*i*_ and *b*_*j*_ are biases for the visible and hidden variables, respectively. *W* ∈ *ℝ*^*c*×*m*^ encodes the weights of connection between *c* visual variables and *m* hidden variables. Then, a joint probability configuration of *v* and *h* can be defined as

(2) $$\begin{array}{c} P\left(\;v,h\;\right)=\frac{\exp\left(-E\left(v,h\right)\right)}{Z}, \end{array}$$

where *Z* is a normalization constant or partition function, *Z* = ∑_*v*,*h*_*e*^−*E*(*v*, *h*)^. The marginal distribution over visible data is

(3) $$\begin{array}{c} P\left(\;v\;\right)=\frac{1}{Z}\underset{h}{\sum }e^{-E\left(v,h\right)}. \end{array}$$

There is no connection between visible units (or hidden units) in an RBM; the conditional distributions over the visible and hidden units are given by logistic functions as follows:

(4) Pvi=1 ∣ h=σai+∑jhjWij

(5) Phi=1 ∣ v=σbj+∑iviWij,

where *σ*(*x*) = 1/(1 + exp⁡(−*x*)) is a logistics sigmoid function.

It is difficult to train an RBM with a large number of parameters. To efficiently train an RBM and to optimize the parameters, we maximize the likelihood of visible data with respect to the parameters. To achieve this goal, the derivative of log probability of the training data derived from (4) can be adopted to incrementally adjust the weights as follows:

(6) $$\begin{array}{c} \frac{\partial \log p\left(v\right)}{\partial W_{ij}}={\langle \;v_{i}h_{j}\;\rangle }_{data}-{\langle \;v_{i}h_{j}\;\rangle }_{model}, \end{array}$$

where 〈·〉 indicates expectations under the distribution. It is very easy to learn the log-likelihood probability of training data:

(7) $$\begin{array}{c} \Delta W_{ij}=\epsilon \left(\;{\langle \;v_{i}h_{j}\;\rangle }_{data}-{\langle \;v_{i}h_{j}\;\rangle }_{model}\;\right), \end{array}$$

where *ϵ* controls the learning rate. Since there are no direct connections in the hidden layer of an RBM, so we can get an unbiased sample of 〈*v*_*i*_*h*_*j*_〉_data_ easily. Unfortunately, it is difficult to compute an unbiased sample of 〈*v*_*i*_*h*_*j*_〉_model_, since it requires exponential time. To avoid this problem, a fast learning algorithm, called Contrastive Divergence (CD) [36], is proposed by Hinton [37]. CD sets visible variables as training data. Then the binary states of hidden units are all computed in parallel using (5). Once the states have been chosen for the hidden units, a “reconstruction” is produced by setting each *v*_*i*_ to 1 with a probability given by (4). In addition, weights are also adjusted in each training pass as follows:

(8) $$\begin{array}{c} \Delta W_{ij}=\epsilon \left(\;{\langle \;v_{i}h_{j}\;\rangle }_{data}-{\langle \;v_{i}h_{j}\;\rangle }_{recon}\;\right). \end{array}$$

〈*v*_*i*_*h*_*j*_〉_data_ is the average value over all input data for each update and 〈*v*_*i*_*h*_*j*_〉_recon_ is the average value over reconstruction; it is considered as a good approximation to 〈*v*_*i*_*h*_*j*_〉_model_.

### 3.2. Deep RBM

**Figure 2:**
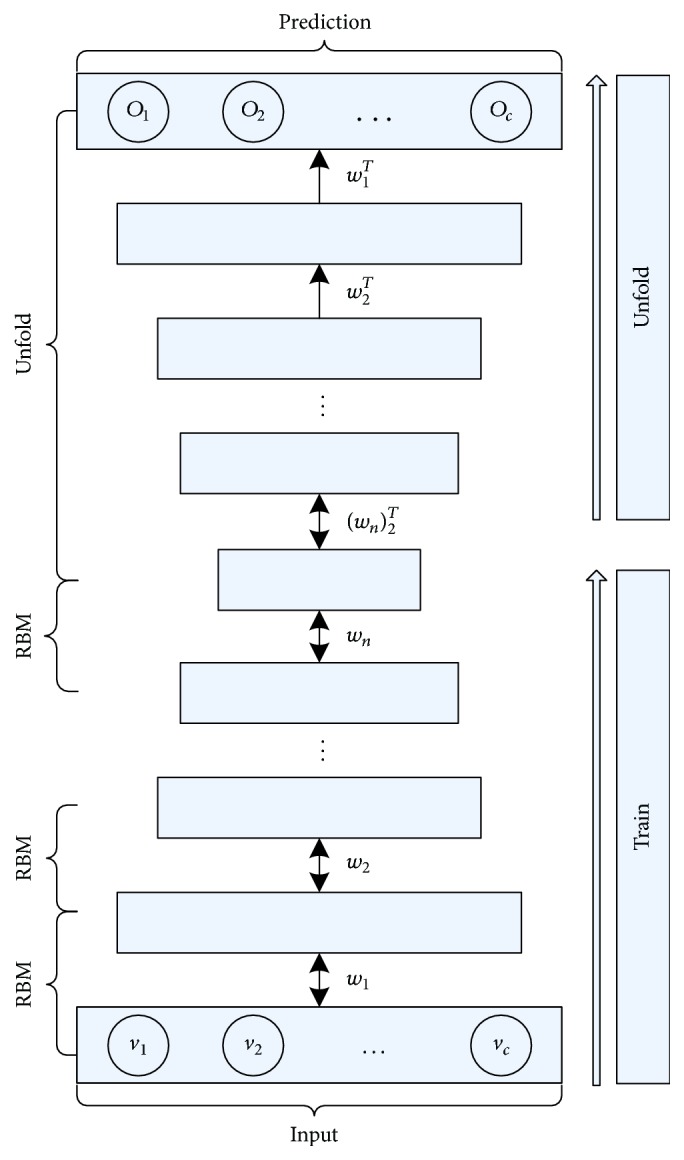
Network architecture of DRBM.

In this paper, we will use a fully connected restricted Boltzmann machine and consider learning a multilayer RBMs (as shown in Figure 2). In the network structure, each layer captures complicated correlations between hidden layer and its beneath layer.

DRBM is adopted for several reasons [38]. Firstly, DRBM, like deep belief networks, has the potential of learning internal representations that become increasingly complex; it is regarded as a promising way to solve complex problems [30]. Second, high-level representations can be built from large volume incomplete sensory inputs and scarce labeled data and then be used to unfold the model. Finally, DRBM can well propagate the uncertainty information and hence robustly deal with ambiguous inputs. Hinton et al. [30] introduced a greedy, layer-by-layer unsupervised learning algorithm that consists of learning a stack of RBMs. After the stacked RBMs have been learned, the whole stack can be viewed as a single probabilistic model. In this paper, we use that greedy algorithm to optimize the parameters of DRBM. DRBM greedily trains a stack of more than two RBMs, and the modification only needs to be used for the first and last RBMs in the stack. Retraining consists of learning a stack of RBMs; each RBM has only one layer of feature detectors. The learned feature activation of one RBM is used as the input data to train the next RBM in the stack. After that, these RBMs are popped up (or unfolded) to create a DRBM. Through the above training, we can optimize the parameters of DRBM and then take the outputs of the network as the results of protein function prediction.

## 4. Result and Discussion

### 4.1. Datasets and Experimental Setup

To study the performance of DRBM on predicting missing GO annotations of incompletely annotated proteins. We downloaded the GO file (http://geneontology.org/page/download-ontology) (archived date: 2015-10-22), which describes hierarchical relationships between GO terms using a DAG. These GO terms are divided into three branches, describing molecular functions (MF), cellular component (CC), and biological process (BP) functions of proteins. We also downloaded the Gene Ontology Annotation (GOA) (archived date: 2014-10-27) files (http://geneontology.org/page/download-annotations) of* Saccharomyces cerevisiae*,* Homo sapiens*,* Mus musculus,* and* Drosophila*. We preprocessed the GO file to exclude the GO terms tagged “obsolete.” To avoid circular prediction, we processed the GOA file to exclude the annotations with evidence code “IEA” (inferred from Electronic Annotation). The missing annotations of a protein often correspond to the descendants of the terms currently annotated to the protein. So the terms corresponding to these missing annotations are located at deeper level than their ancestor terms, and these terms characterize more specific biological functions of proteins than their ancestors. These specific terms are usually annotated to no more than 30 proteins; they are regarded as sparse functions. On the other hand, root terms, GO:0008150 for BP, GO:0003674 for MF, and GO:0005575 for CC, are annotated to majority of proteins; the prediction on these terms is not interesting, so we removed these three root terms. We kept the terms annotated at least one protein in the GOA file for experiments. The statistics of preprocessed GO annotations of proteins in these four model species are listed in Table 1.

We also downloaded recently archived GOA files (date: 2015-10-12) of these four species to validate the performance of DRBM and processed these GOA files in a similar way. We use the data archived in 2014 to train DRBM and then use the data archived in 2015 for validation.

In order to comparatively evaluate the performance of DRBM, we compare it with SVD [15], NtN [16], dRW [18], and AE [29]. SVD, NtN, and dRW are shallow machine learning algorithms. AE and DRBM are deep machine learning methods. DRBM is set with a learning rate of 0.01 for 25 iterations [29]. *L*2 regularization is used on all weights, which are initialized randomly from the uniform distribution between 0 and 1. We set the hidden unit function as sigmoid and the number of hidden units as half of visible units and the number of the second hidden layer as half of the first hidden layer and so on. The number of hidden layers is 5. In the following experiments, to prevent overfitting, we used weight-decay and dropout. Weight-decay adds an extra term to the normal gradient. This extra term is the derivative of a function that penalizes large weights. We used the simplest *L*2 penalty function. As well as that, dropout is a regularization technique for reducing overfitting in neural networks by preventing complex coadaptations on training data [39].

The accuracy of protein function prediction can be evaluated by different evaluation metrics, and the performance of different prediction models is affected by the adopted evaluation metrics. To do a fair and comprehensive comparison, we used four evaluation metrics, *MacroAvgF*1, *AvgROC*, *RankingLoss*, and *Fmax*. These evaluation metrics measure the performance of protein function prediction from different aspects. The first three metrics have been applied to evaluate the results of multilabel learning [40]. *AvgROC* and *Fmax* are recommended metrics for evaluating protein function prediction [6, 41]. *MacroAvgF*1 gets the *F*1-Score of each term and then takes the average of *F*1-score across all the terms. *AvgAUC* firstly calculates the area under receiver operating curve of each term and then takes the average value of these areas as whole to measure the performance. *Fmax* [6] is the overall maximum harmonic mean of recall and precision across all possible thresholds on the predicted protein function association matrix. *RankingLoss* computes the average fraction of wrongly predicted annotations ranking ahead of ground-truth annotations of proteins. To be consistent with other evaluation metrics, we use 1 − *RankLoss* instead of *RankingLoss*. Namely, the higher the value of these metrics is, the better the performance is. The formal definition of these metrics can be found in [6, 22, 40]. Since these metrics capture different aspects of a function prediction method, it is difficult for an approach to consistently outperform the others across all the evaluation metrics.

### 4.2. Experimental Results

Based on the experimental protocols introduced above, we conduct experiments to investigate the performance of DRBM on protein function prediction. In Table 2, we report the experimental results on proteins of* Homo sapiens* annotated with BP, CC, and MF terms, respectively. The results on* Mus musculus*,* Saccharomyces cerevisiae*, and* Drosophila* are provided in Tables 3–5. In these tables, the best results are in* boldface*.

From these tables, we can see that DRBM achieves better results than NtN, dRW, SVD, and AE in most cases. We further analyze the differences between DRBM and these comparing methods by Wilcoxon signed rank test [42, 43], we find that DRBM performs significantly better than NtN, dRW, and SVD on the first three metrics (where *p* values are all smaller than 0.004), and it also gets better performance than deep AE across these four metrics (*p* value smaller than 0.001). dRW often obtains larger *F*max than DRBM; the possible reason is that dRW utilizes threshold to filter out some predictions and thus increases the true positive rate.

dRW applies downward random walks with restart on the GO directed acyclic graph to predict protein function; dRW takes into count the hierarchical structure relationship between GO terms and achieves better results than NtN and SVD. This observation confirms that the hierarchical relationship between terms plays important roles in protein function prediction. Although dRW utilizes the hierarchical structure relationship between terms, it is still a shallow machine learning method and it does not capture the deep associations between proteins and GO terms as DRBM does, so it is often outperformed by DRBM.

The results of NtN and SVD are always lower than those of AE and DRBM. The possible reason is that singular value decomposition on sparse matrix is not suitable for this kind of protein function prediction problem, in which there are complex hierarchical relationships between GO terms. NtN uses the ontology hierarchy to adjust the weights of protein function associations, but it does not get better results than SVD. The reason is that NtN gives large weights to specific annotations but small weights to shallow annotations. From the true path rule, ancestor terms are generally annotated to more proteins than their descendant terms. For this reason, NtN is often outperformed by SVD and say nothing of AE and DRBM. Both AE and DRBM are deep machine learning techniques, but DRBM frequently performs better than AE. That is because the generalization ability of AE is not as well as that of DRBM, and AE is easy to fall into local optimal. In summary, these results and comparisons demonstrate that DRBM can capture deep associations between proteins and GO terms, and thus it achieves better performance than other related methods across different evaluation measures. DRBM is an effective alternative approach for protein function prediction.

### 4.3. Runtime Analysis

Here, we study runtime (include training phase and test phase) cost of these comparing methods on* Homo sapiens* and* Mus musculus* in GO BP subontology, since this subontology includes much more annotations and GO terms. The experimental platform is Windows Server 2008, Intel Xeon E7-4820, 64 GB RAM. The recorded runtime for these comparing methods is reported in Table 6.

From this table, we can see that DRBM is faster than these comparing methods, except SVD. NtN and dRW spend a lot of time to compute semantic similarity between GO terms, so they take more time than others. In contrast, SVD directly applies matrix decomposition on the protein function association matrix and the matrix is sparse, so SVD takes fewer time than DRBM. AE employs back propagation neural networks to tune parameters; it costs a large amount of time. DRBM utilizes Contrastive Divergence, which is a fast learning algorithm, to optimize the parameters, so its runtime is fewer than AE. This comparison further confirms DRBM is an efficient and effective alternative solution for protein function prediction.

## 5. Conclusions

In this paper, we study how to predict additional functional annotations of annotated proteins. We investigate deep restricted Boltzmann machines (DRBM) for this purpose. Our empirical study on the proteins of* Saccharomyces cerevisiae*,* Homo sapiens*,* Mus musculus*, and* Drosophila* shows that DRBM outperforms several competitive related methods, especially shallow machine learning models. This paper will drive more research on using deep machine learning techniques for protein function prediction. As part of our future work, we will integrate other types of proteomic data with DRBM to further boost the prediction performance.

## Figures and Tables

**Table 1:** Statistics of experimental datasets. The data in the third column (*N*) is the number of proteins annotated with at least 1 term for a particular subontology. *C* is the number of involved GO terms; Avg ± Std is the average number of annotations of a protein and its standard deviation.

Dataset		*N*	*C*	Avg ± Std
*Homo sapiens*	BP	11628	12514	60.24 ± 60.83
	CC	12523	1574	20.17 ± 12.28
	MF	11628	3724	10.97 ± 8.81

*Mus musculus*	BP	10990	13500	56.26 ± 61.08
	CC	10549	1592	15.73 ± 10.25
	MF	9906	3775	9.59 ± 7.30

*Saccharomyces cerevisiae*	BP	4671	4909	44.13 ± 31.41
	CC	4128	970	20.67 ± 10.30
	MF	4291	2203	9.60 ± 6.60

*Drosophila*	BP	6188	6645	48.53 ± 48.97
	CC	4851	1097	15.10 ± 10.27
	MF	4489	2255	9.05 ± 5.75

**Table 2:** Experimental results on *Homo sapiens*.

		MacroAvg*F*1	AvgROC	1 − RankLoss	*F*max
BP	NtN	0.0107	0.7498	0.6920	0.1712
	dRW	0.6902	0.9044	0.8737	**0.9301**
	SVD	0.7313	0.9053	0.9349	0.9206
	AE	0.5341	0.9049	0.8495	0.5617
	DRBM	**0.8378**	**0.9109**	**0.9883**	0.9217

CC	NtN	0.0036	0.6569	0.6641	0.1063
	dRW	0.6806	0.8999	0.9186	**0.9516**
	SVD	0.7139	0.8942	0.9592	0.9157
	AE	**0.8081**	0.8932	0.9629	0.8819
	DRBM	0.7982	**0.9192**	**0.9955**	0.9437

MF	NtN	0.3891	0.7767	0.8450	0.0121
	dRW	0.7909	**0.9130**	0.9208	**0.9529**
	SVD	0.8022	0.8022	0.9526	0.9480
	AE	0.7683	0.9047	0.8186	0.5604
	DRBM	**0.8517**	0.9085	**0.9898**	0.9470

**Table 3:** Experimental results on *Mus musculus*.

		MacroAvg*F*1	AvgROC	1 − RankLoss	*F*max
BP	NtN	0.0154	0.6950	0.7055	0.1542
	dRW	0.5666	0.8155	0.8296	**0.9049**
	SVD	0.6169	0.8220	0.9130	0.8914
	AE	0.4573	0.8139	0.8219	0.5340
	DRBM	**0.7221**	**0.8476**	**0.9841**	0.8962

CC	NtN	0.0055	0.6244	0.6436	0.1062
	dRW	0.4913	0.8001	0.7857	**0.8694**
	SVD	0.5415	0.7847	0.8856	0.8539
	AE	0.6548	0.7933	0.9139	**0.8694**
	DRBM	**0.6676**	**0.8412**	**0.9813**	0.8644

MF	NtN	0.7338	0.9135	0.9401	0.0111
	dRW	0.8742	0.9493	0.9474	**0.9693**
	SVD	0.7408	0.9466	0.9703	0.9188
	AE	0.9035	0.9461	0.9724	0.7044
	DRBM	**0.9133**	**0.9492**	**0.9906**	0.9652

**Table 4:** Experimental results on *Saccharomyces cerevisiae*.

		MacroAvg*F*1	AvgROC	1 − RankLoss	*F*max
BP	NtN	0.0072	0.7026	0.7027	0.1172
	dRW	0.8042	**0.9268**	0.9337	**0.9649**
	SVD	0.7794	0.9199	0.9659	0.9440
	AE	0.6990	0.9179	0.9252	0.5032
	DRBM	**0.8524**	0.9256	**0.9905**	0.9555

CC	NtN	0.0072	0.7026	0.7027	0.1172
	dRW	0.8112	0.9264	0.9612	**0.9771**
	SVD	0.7408	0.9274	0.9767	0.9198
	AE	0.8595	0.9262	0.9851	**0.9771**
	DRBM	**0.8722**	**0.9278**	**0.9948**	0.9744

MF	NtN	0.7338	0.9135	0.9401	0.0111
	dRW	0.8742	**0.9493**	0.9474	**0.9693**
	SVD	0.7408	0.9466	0.9703	0.9188
	AE	0.9035	0.9461	0.9724	0.7044
	DRBM	**0.9133**	0.9492	**0.9906**	0.9652

**Table 5:** Experimental results on *Drosophila*.

		MacroAvg*F*1	AvgROC	1 − RankLoss	*F*max
BP	NtN	**0.7724**	0.8450	0.8958	0.9416
	dRW	0.6875	0.8525	0.9011	**0.9455**
	SVD	0.6852	0.8516	0.9479	0.9371
	AE	0.5882	0.8486	0.9049	0.5772
	DRBM	0.7699	**0.8601**	**0.9877**	0.9382

CC	NtN	0.0101	0.6475	0.7808	0.1957
	dRW	0.6599	0.8425	0.9210	**0.9553**
	SVD	0.6446	0.8222	0.9585	0.9156
	AE	0.7331	0.8251	0.9678	**0.9553**
	DRBM	**0.7438**	**0.8558**	**0.9922**	0.9448

MF	NtN	0.5071	0.7640	0.9065	0.0700
	dRW	0.7346	**0.8206**	0.9309	**0.9610**
	SVD	0.7131	0.8125	0.9631	0.9549
	AE	0.7558	0.8133	0.9639	0.6429
	DRBM	**0.7719**	0.8187	**0.9895**	0.9499

**Table 6:** Runtime cost (seconds) on *Homo sapiens* and *Mus musculus* in BP subontology.

	NtN	dRW	SVD	AE	DRBM
*Homo sapiens *	30180	27660	1200	15840	6180
*Mus musculus *	24180	28020	1260	33780	7500
